# Sick Sinus Syndrome Mimicking Autonomic Dysfunction of Dementia With Lewy Bodies

**DOI:** 10.7759/cureus.14667

**Published:** 2021-04-24

**Authors:** Ryo Sawagashira, Yoshihisa Sasagawa, Mayumi Matsukura, Yuji Takamaru

**Affiliations:** 1 Physiology, Hokkaido University, Sapporo, JPN; 2 Psychiatry, Hokkaido University, Sapporo, JPN; 3 Psychiatry, Otaru General Hospital, Otaru, JPN

**Keywords:** dementia with lewy bodies, autonomic dysfunction, sick sinus syndrome, pacemaker implantation, delirium, donepezil

## Abstract

Dementia with Lewy bodies (DLB) is recognized as the second most common form of dementia in aged people. It is well known that patients with DLB often develop various autonomic symptoms. Here, we present a case in which there was sick sinus syndrome mimicking the DLB-related autonomic dysfunctions. After the pacemaker implantation, the patient’s symptom perfectly extinguished.* *It is essential for psychiatrists or other professionals who are mainly seeing dementia patients to rule out critical causes that may mimic autonomic symptoms in patients with DLB.

## Introduction

Dementia with Lewy bodies (DLB) is currently recognized as the second most common form of dementia in aged people from 50 to 70 years old, accounting for around 15%-20% cases at autopsy [1]. Clinically, DLB can be diagnosed by the presence of progressive cognitive decline accompanied by two out of four of the following core features; fluctuating cognition, recurrent visual hallucinations, spontaneous motor parkinsonism, and rapid eye movement (REM) sleep behavior disorder [2]. Moreover, it is well reported that most DLB cases present autonomic dysfunctions such as dizziness, orthostatic hypotension, urinary incontinence, constipation, and sweat deregulation. However, various other causes like sick sinus syndrome develop dizziness in elderly people. Here, we present a case of an 84-year-old female with a sick sinus syndrome mimicking DLB-related autonomic dysfunctions. Following pacemaker implantation, the patient experienced improvements both in her autonomic and psychiatric symptoms.

## Case presentation

An 84-year-old female was independent one year ago without past medical history. Two months before admission, the patient had developed a short memory span and difficulties walking. About two weeks before admission, she developed visual hallucination, complaining repeatedly that she could see a thief entering her house every day at midnight. In contrast, the patient spent her daytime calmly and never complained of such sightings. Her family doctor clinically diagnosed her with probable DLB with fluctuating cognition, recurrent visual hallucinations and spontaneous motor parkinsonism. She received 5 mg per day of donepezil around the same time. About one week before admission, her psychiatric symptoms deteriorated and she started calling the police almost every night. She was admitted to our psychiatric ward due to worsening of visual hallucination and insomnia.

**Figure 1:**
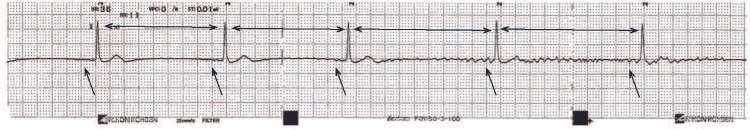
Monitoring electrocardiogram findings P-waves were not detected, and RR intervals were significantly prolonged (>1.6 seconds).

After admission, she continued to complain repeatedly of seeing a thief entering her room in addition to dizziness and sweat deregulation, which were thought to be DLB-related autonomic dysfunctions. Single-photon emission tomography (SPECT) scan detected a significant decrease in cerebral blood flow in the occipital area and this finding supported the diagnosis of DLB. We prescribed 4 mg of the antipsychotic perospirone with a relatively short half-life in order to manage her psychiatric symptoms, paying close attention to hypersensitivity for antipsychotics. However, the symptoms did not get better and her insomnia persisted. On day 20 after admission, she suddenly developed a loss of consciousness while sitting, which triggered us to perform an electrocardiogram (ECG) examination (Figure 1).

The findings revealed severe bradycardia, prompting us to consult cardiologists. Subsequently, the patient was immediately subjected to Holter monitoring in order to detect the origins of syncope. The findings disclosed the cardiac arrest of approximately 7 seconds, and the patient was diagnosed with sick sinus syndrome. Finally, she was transferred to the cardiac ward and had pacemaker implantation. After she was transferred again to our psychiatric ward on day 29 after admission, dizziness and loss of consciousness perfectly disappeared. However, visual hallucination, insomnia, and sweat deregulation persisted. Thus, we again prescribed 4 mg of perospirone, not donepezil. From day 50 onwards after admission, her visual hallucinations and insomnia gradually decreased and she spent her time more calmly without any side effects of antipsychotics.

## Discussion

In our case, an 84-year-old patient with DLB presented with dizziness and deregulation of sweat on admission. At first, we thought of those symptoms as part of typical DLB-related autonomic dysfunctions because she had no past medical history, and laboratory data and ECG were within normal limits on admission. However, we suspected other critical causes existing in the background of some autonomic dysfunctions (dizziness) because the patient lost her consciousness while sitting on day 20 after admission. Finally, we determined sick sinus syndrome was the cause of her loss of consciousness. After implantation of a pacemaker, loss of consciousness and dizziness perfectly improved, but deregulation of sweat persisted. Finally, her psychiatric conditions also got improved with a small dose of antipsychotics without any side effects.

Sick sinus syndrome is a collection of disorders defined by abnormal cardiac impulse formation and abnormal propagation from the sinoatrial node, which impair its pacemaking function. One in 600 patients with cardiac disease older than 65 years presents this syndrome. In one study of patients older than 21 years with sick sinus syndrome, the median age was 74 years. Sick sinus syndrome affects men and women equally, and its etiology can be divided into intrinsic and extrinsic factors that disrupt the function of the sinoatrial node [3].

In our case, several factors were suspected to be the cause of sick sinus syndrome. Aging is one intrinsic factor that may cause sick sinus syndrome. Extrinsic factors that can cause or exacerbate the sick sinus syndrome include the use of certain pharmacologic agents, metabolic disturbances, and autonomic dysfunctions. The pharmacologic agents that commonly cause dysfunction of the sinoatrial node are beta-blockers, calcium channel blockers, digoxin, sympatholytic medications, antiarrhythmic medications, and lithium. In our case, donepezil was administered for two weeks before admission and might be a candidate cause of sick sinus syndrome. In a previous study from 2005, Bordier et al. [4] examined patients with Alzheimer’s disease who were treated with donepezil and experienced syncope and determined the cause of the syncope in 69% of patients. They found complete atrioventricular blockade in two cases, carotid sinus disease in three, sinus node dysfunction in two, severe orthostatic hypotension in two, and paroxysmal atrial fibrillation in one [4]. Although our patient’s family doctor checked her ECG regularly and confirmed normality, we could not rule out the possibility of donepezil-induced sick sinus syndrome. On the other hand, autonomic dysfunction can cause or exacerbate sick sinus syndrome via neurally mediated bradycardia in the vasovagal syncope, neurocardiogenic syncope, and carotid sinus hypersensitivity [4]. It is reported that multiple system atrophy, which is a neurodegenerative disorder included in α-synucleinopathies like DLB, may cause respiratory arrest due to autonomic dysfunction [5]. Moreover, it is reported that patients with diabetes mellitus and autonomic disturbances have a high risk of sudden death due to cardiac arrest [6]. Thus, it is plausible that autonomic dysfunction itself directly causes sick sinus syndrome in patients with DLB.

Finally, in our case, implantation of a pacemaker improved not only the autonomic symptoms but also psychiatric symptoms. The relationship between DLB psychiatric symptoms and delirium may be useful to explain the progress of our case.

Delirium is a complex neuropsychiatric condition of acute brain dysfunction with multifactorial etiology. It is common in hospitalized older patients with 10%-31% prevalence at admission and 11%-42% incidence rates. The presentation of delirium is highly variable; disturbances in attention and awareness are central to the diagnosis, with other common abnormalities including abnormal motor behaviors, sleep-wake cycle abnormalities, in addition to disturbances in emotion, perception, and thinking. Delirium is much more common in patients with dementia or chronic cognitive impairments. A number of clinical features are shared by DLB and delirium, making the distinction between the two difficult, particularly at the initial stages of presentation [7]. In summary, the relationship between DLB psychiatric symptoms and delirium remains vague and the two symptoms are not clearly distinguished. In our case, implantation of a pacemaker improved the patient’s physical condition, which was followed by amelioration of a part of her psychiatric symptoms.

## Conclusions

We presented a case of an 84-year-old female with a sick sinus syndrome mimicking DLB-related autonomic dysfunctions. Following pacemaker implantation, the patient experienced improvements both in her autonomic and psychiatric symptoms. Clinicians should distinguish autonomic dysfunctions that originated from DLB itself and those from other causes.
